# Controllable Vapor Growth of Large-Area Aligned CdS_*x*_Se_1−*x*_ Nanowires for Visible Range Integratable Photodetectors

**DOI:** 10.1007/s40820-018-0211-7

**Published:** 2018-06-23

**Authors:** Muhammad Shoaib, Xiaoxia Wang, Xuehong Zhang, Qinglin Zhang, Anlian Pan

**Affiliations:** Key Laboratory for Micro-Nano Physics and Technology of Hunan Province, State Key Laboratory of Chemo/Biosensing and Chemometrics, College of Materials Science and Engineering, Hunan University, Changsha, 410082 Hunan People’s Republic of China

**Keywords:** Graphoepitaxial effect, Bandgap engineering, CdS_*x*_Se_1−*x*_ nanowires, Optical waveguide, Photodetectors

## Abstract

The controllable growth of large area band gap engineered-semiconductor nanowires (NWs) with precise orientation and position is of immense significance in the development of integrated optoelectronic devices. In this study, we have achieved large area in-plane-aligned CdS_*x*_Se_1−*x*_ nanowires via chemical vapor deposition method. The orientation and position of the alloyed CdS_*x*_Se_1−*x*_ NWs could be controlled well by the graphoepitaxial effect and the patterns of Au catalyst. Microstructure characterizations of these as-grown samples reveal that the aligned CdS_*x*_Se_1−*x*_ NWs possess smooth surface and uniform diameter. The aligned CdS_*x*_Se_1−*x*_ NWs have strong photoluminescence and high-quality optical waveguide emission covering almost the entire visible wavelength range. Furthermore, photodetectors were constructed based on individual alloyed CdS_*x*_Se_1−*x*_ NWs. These devices exhibit high performance and fast response speed with photoresponsivity ~ 670 A W^−1^ and photoresponse time ~ 76 ms. Present work provides a straightforward way to realize in-plane aligned bandgap engineering in semiconductor NWs for the development of large area NW arrays, which exhibit promising applications in future optoelectronic integrated circuits.

**Electronic supplementary material:**

The online version of this article (10.1007/s40820-018-0211-7) contains supplementary material, which is available to authorized users.

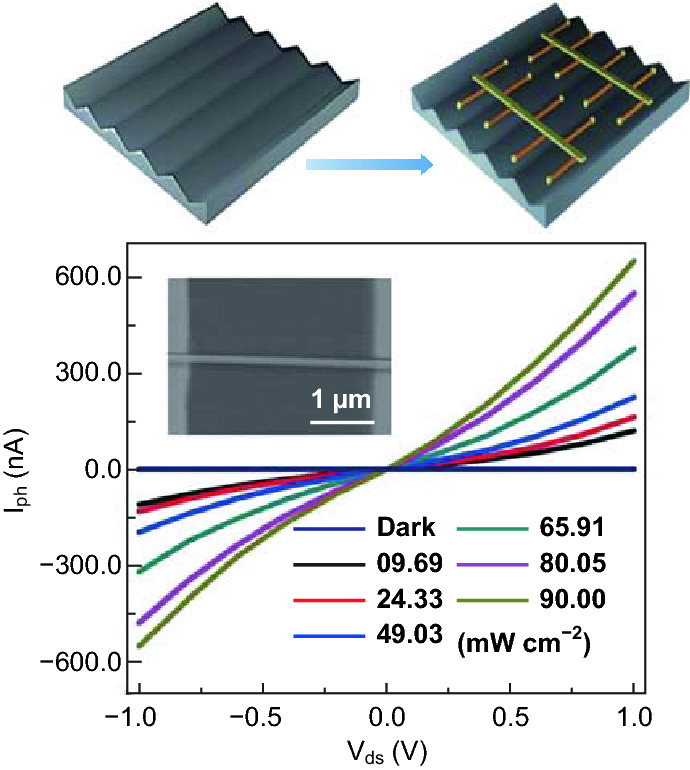

## Highlights

- The growth of tunable composition-directional CdS_*x*_Se_1−*x*_ nanowires was successfully realized by controllable chemical vapor deposition using graphoepitaxial effect.
- Photodetectors based on CdS_*x*_Se_1−*x*_ nanowires with different compositions covering the visible spectral range on faceted *M*-plane substrate were constructed.
- The as-grown nanowires not only exhibited superior optical properties such as strong emission and perfectly aligned waveguide but also demonstrated high-performance photodetection as compared to previous single crystalline CdSSe photodetectors.

## Introduction

One-dimensional semiconductor nanowires (NWs) have stimulated enormous attention among researchers owing to their excellent optical characteristics and remarkable optoelectronic applications such as light-emitting diodes, lasers, sensors, and photodetectors (PDs) [1–10]. In particular, wide-bandgap semiconductor NWs such as CdS, CdSe, ZnO, and ZnS have been demonstrated to be suitable for high-performance optoelectronic devices owing to their high photochemical stability and remarkable optical properties [11–17]. However, intrinsic bandgap of these binary semiconductors can hardly be tuned, which inevitably limits their further applications in photonic and optoelectronic devices [18, 19]. In recent years, ternary or multicomponent-alloyed semiconductor NWs such as those of Zn_*x*_Cd_1−*x*_S, Zn_*x*_ Cd_1−*x*_Se, and ZnCdSSe have extended the type of applications because their bandgap energy can be modulated by altering their elemental compositions [20–25]. CdSe nanowire PDs show a larger photoresponse because of production of more photo-generated carriers owing to its narrower bandgap (1.74 eV) compared to that of other compositions of the CdSSe NWs. The absorption spectrum of CdSSe NWs covers almost whole of the visible solar radiation range making it suitable for a wider photodetecting wavelength. More importantly, single crystalline, ternary alloyed CdS_*x*_Se_1−*x*_ semiconductor NWs possess outstanding architecture to provide engineered bandgap superiority that is essential for the development of broadband response optoelectronic devices to be functional in the visible range [26–31].

Several approaches have been developed to tune the composition to obtain free-standing CdSSe nanostructures; our group especially has reported several studies for the realization of alloyed CdS_*x*_Se_1−*x*_ NWs used in high-performance broadband lasers and PDs by a home-built multistep thermal evaporation route with a moving source equipment [32–36]. However, to achieve high-density integration photonics systems, large scale horizontally aligned NW arrays are required, which are critical for next-generation optoelectronic integration devices [37, 38]. In recent years, Ernesto Joselevich and co-workers have demonstrated guided growth of binary semiconductors NWs (e.g., CdS, ZnO and GaN) on a sapphire substrate to control their alignment and position for developing promising optoelectronic devices [37, 39–43]. To this end, graphoepitaxial effect specifically leads the semiconductor NWs along selective in-plane directions. However, to the best of our knowledge, synthesis of bandgap engineered in-plane directional CdS_*x*_Se_1−*x*_ NWs with precise orientation and position is still a challenge. This work shows a high-quality ternary CdS_*x*_Se_1−*x*_ NWs system used for high-performance PDs, expands the family of semiconductor materials for high-performance visible PD by guided growth technique, and offers a new procedure to modulate the bandgap by tuning the atomic ratios of ternary wurtzite semiconducting materials.

In this paper, we report for the first time, the synthesis of single crystal-alloyed, in-plane-aligned CdS_*x*_Se_1−*x*_ NWs via a simple one-step physical evaporation process on annealed *M*-plane sapphire. The orientation and the position of the NWs are controlled well by the graphoepitaxial effect and the pre-deposited Au catalyst pattern. The as-grown nanowires are well-aligned, with uniform diameter and smooth surface. Moreover, tunable photoluminescence (PL) emissions and optical waveguide behavior from green (510 nm) to red (710 nm) wavelength was detected using these alloyed CdS_*x*_Se_1−*x*_ NWs which almost covers the entire visible range. In addition, the PD properties of pure CdS, CdSe, and CdS_*x*_Se_1−*x*_ directional NWs were systematically investigated with high photoresponsivity (~ 670 A W^−1^) and fast response speed (~ 76 ms). This work may pave the way to explore other alloy semiconductor materials for realizing broad spectral response PDs and lasing behavior in the field of integrated photonic circuits in the visible regime.

## Experimental Section

*M*-plane sapphire was annealed at 1400 °C for 10 h in ambient atmosphere. Prior to use, the substrates were sonicated for 10 min each in acetone, isopropyl alcohol (IPA), and distilled H_2_O, and then blow-dried in N_2_. After annealing, patterns of different sizes such as 500 × 500 nm^2^ by EBL, 100 × 5 µm^2^ and 10 × 5 µm^2^ by photolithography and 100 µm apart were designed perpendicular to the growth direction of nanowires. Furthermore, Au catalyst was deposited by electron beam evaporation of a thin (10 nm) Au film.

The thin Au film was heated under 800 °C for 15 min, thus generating the nanoparticles that serve as catalyst for the vapor–liquid–solid (VLS) growth of NWs. High-purity N_2_ gas was introduced into the quartz tube at a constant flowing rate (100 sccm) for 60 min, to purge the O_2_ inside. After 30 min, the furnace was rapidly heated to 700 °C and maintained at 50 torr pressure for 30 min. To prepare the ternary CdS_*x*_Se_1−*x*_ NWs with different compositions, mixture of CdS and CdSe powders was placed at the center of the heating zone, the stoichiometry of the alloys was readily controlled by adjusting the S:Se molar ratio of the source powder as shown in Fig. S5. CdS and CdSe directional NWs were prepared using the same procedure, except that only CdS or CdSe powder was used as the evaporation source at the deposition temperature of 500–550 °C. After the growth, bulk of the catalyst deposited area was covered with vertically aligned NWs, whereas a large number of horizontally aligned NWs extended onto the clean sapphire surface. After sonication for a few seconds, the vertically aligned NWs were removed, leaving only the horizontal NWs in the sapphire substrate.

For the construction of PDs, we used electron beam lithography for the individual-guided NWs (diameter 100 nm) and then deposited Cr/Au (10 nm/60 nm) electrodes by electron beam evaporation. The optoelectronic properties of the as-fabricated PDs were investigated by exploring their photocurrent generation, photoresponsivity, quantum efficiency, stability, and response speed at room temperature.

## Results and Discussion

**Fig. 1:**
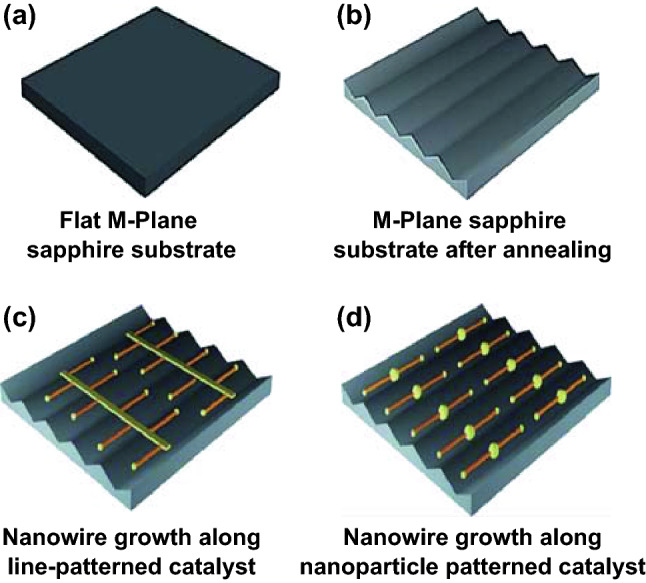
Growth schematic for the in-plane-directional CdS_*x*_Se_1−*x*_ NWs. **a** *M*-plane sapphire substrate surface before annealing. **b** *M*-plane substrate surface with *V*-shape grooves after high temperature annealing. **c**–**d** VLS growth of NWS along the pre-defined Au catalyst patterns

Figure 1 illustrates the schematic of the growth progression for the guided CdS_*x*_Se_1−*x*_ NWs on annealed *M*-plane sapphire substrate, which has a flat surface as shown in Figs. 1a and S1a. However, *V*-shaped nanogroove structures formed on the substrate surface after high-temperature annealing as shown in Figs. 1b and S1b. It is because *M*-plane sapphire is thermodynamically unstable at high temperatures. Its crystal facet is transformed from $\left( {10\overline{{10}} } \right)$ to nanostructure *V*-shape nanogrooves composed of S (1011) and *R* (1102) facets during high-temperature annealing [44]. The existence of the grooves in our study is consistent with the observations in other reported guided growth semiconductor NW systems and the same mechanism would be applied for the growth of parallel CdS_*x*_Se_1−*x*_ NWs on the annealed *M*-plane substrate as well.

To realize the direction and position for the aligned NWs during controllable VLS growth, we deposited Au catalyst with different patterns. Figure 1c shows schematically line-patterned Au catalyst, whereas Fig. 1d shows the Au catalyst nanoparticles, patterned with precise position, deposited by electron beam lithography (EBL). The in-plane well-aligned CdS_*x*_Se_1−*x*_ NWs were synthesized by a previously reported simple chemical vapor deposition route. The faceted surface of *M*-plane substrate plays an important role in the initial nucleation of the Au catalyst and subsequent growth of a NW on the surface. At the first step, gold particles act as preferential sink to collect material from the surrounding vapor reactants and the wires are formed spontaneously as a strain relieving graphoepitaxial effect. Next, the nanogrooves continuously exert a strong anisotropic force on precursors at nucleation sites to grow longer CdS_*x*_Se_1−*x*_ NWs along the nanogrooves, as shown schematically in Fig. 1c–d. In general, on the faceted surface of *M*-plane sapphire substrate, the graphoepitaxial effect controls the growth direction of the NWs along the *V*-shaped nanogrooves.

**Fig. 2:**
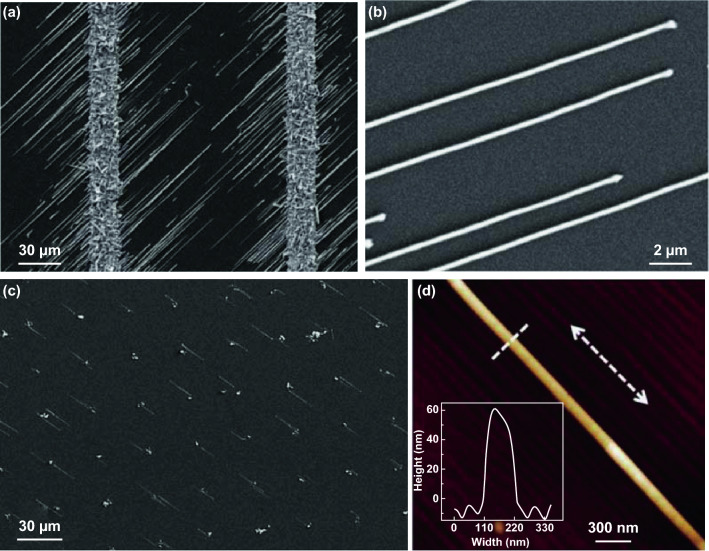
Morphology characterization of the directional CdS_*x*_Se_1−*x*_ NWs. **a** SEM images of the as-grown CdS_*x*_Se_1−*x*_ NWs along the pre-defined photolithography line-patterned Au catalyst. **b** High-magnification SEM images of the as-grown samples, showing strong horizontal alignment of the NWs along the substrate. **c** SEM images of the CdS_*x*_Se_1−*x*_ NWs grown along the pre-defined catalytic nanoparticles by EBL. **d** AFM images of the directional CdS_*x*_Se_1−*x*_ NWs, inset shows the size profile of the NW of the dashed line marked position in (**d**)

The morphology of the as-grown samples on the *M*-plane sapphire substrate with different Au catalyst patterns was first characterized using scanning electron miscroscopy (SEM) and atomic force microscopy (AFM). Figures 2a and S1 present the SEM images of the CdS_*x*_Se_1−*x*_ NW arrays showing that the large area nanowires with high density, good uniformity, and well-defined morphology are strongly oriented on the substrate. Typical high-magnification SEM image of the directional CdS_*x*_Se_1−*x*_ NWs reveals that the NWs have uniform diameter and smooth surface, as depicted in Fig. 2b. The corresponding lengths and diameters of the as-grown NWs ranged from 50 to 100 µm and 80 to 150 nm, respectively, and could be controlled by the size of catalyst and growth time. The homogeneous elemental composition of these directional CdS_*x*_Se_1−*x*_ NWs because of the substitution of Se^2−^ by S^2−^, leads to a smooth shift from pure CdS to CdSe, primarily attributed to their good lattice parameter matching (Fig. S2). These results reveal that the CdS_*x*_Se_1−*x*_ NWs with different compositions are not crucial for the graphoepitaxial effect that provides minimum surface-free energy for the interface between the semiconductor and the metal catalyst to grow NWs preferentially along *V*-shaped grooves, in agreement with the growth mechanism to that of reported well-aligned NWs [36, 37].

We achieved high-density well-defined uniform NWs along nanoparticle Au catalyst patterns, as shown in Fig. 2c. It was found that the nanowires grow along bidirectional orientation due to the existence of nanogrooves, which is quite similar to the observed orientation growth of CdS nanorods (NRs) and NWs on faceted *M*-plane substrate [40]. Figure 2d shows the AFM image of a representative directional NW within the nanogroove along the faceted surface of the substrate. The inset shows the size profile of the NW with a diameter of 60 nm and depth of the V-groove was measured to be 12 nm, as shown in Fig. S1c, d, which also indicates the NW well-oriented along the nanogrooves because of graphoepitaxial effect. To investigate the crystal structure, we implemented X-ray diffraction (XRD) for pure directional CdS NWs (Fig. S3). The strong diffraction (101) peaks, together with the weaker (100) and (002) peaks could be indexed to the wurtzite phase of pure CdS NWs. These results demonstrate that large area in-plane-aligned CdS_*x*_Se_1−*x*_ NWs with well-defined morphology and smooth surface were successfully achieved on the annealed *M*-plane sapphire substrates.

**Fig. 3:**
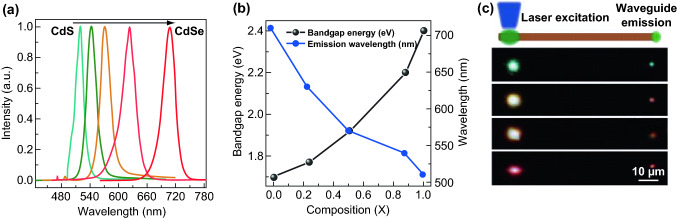
Optical properties of the in-plane-directional CdS_*x*_Se_1−*x*_ NWs. **a** Tunable PL spectra of the as-grown CdS_*x*_Se_1−*x*_ NWs with the emission wavelength shifts from 510 nm (pure CdS) to 710 nm (pure CdSe). **b** Composition-dependent bandgap energy and the corresponding emission wavelength of the as-grown CdS_*x*_Se_1−*x*_ NWs. **c** The schematic diagram of the CdS_*x*_Se_1−*x*_ NW waveguide and the corresponding real-color images of single CdS, CdS_0.8_Se_0.2_, CdS_0.24_Se_0.76_, and CdSe NWs, respectively

Optical emission properties of the as-grown directional CdS_*x*_Se_1−*x*_ NWs were examined at room temperature. Figure 3a shows the normalized PL spectra of band-engineered CdS_*x*_Se_1−*x*_ NWs under the illumination of focused laser. Each composition showed a high-quality band-edge emission spectrum with the peaks continuously shifting from 510 nm (for pure CdS) to 710 nm (for pure CdSe). In general, it is known that the band gap of a ternary alloy is determined by an interpolation between those of the two binaries with additional nonlinear bowing for CdSe_*x*_S_1−*x*_ and the band gap bowing parameter (b: 0.54) [45]. Therefore, we can calculate the band gap energy of each S-molar fraction using the following equation [46, 47].

$$E_{g} (CdS_{x} Se_{1 - x} ) \, = xE_{g} \left( {CdS} \right) + \left( {1 - x} \right)E_{g} \left( {CdSe} \right) - x\left( {1 - x} \right)b$$

Based on the calculation, we further plot S-molar fraction *x*-dependent emission wavelength and the bandgap energy as in Fig. 3b; the plot shows well-modulated behavior as the composition *x* varies from 0 to 1. The continuous shifting of the emission wavelength for the obtained NWs gives further evidence for the formation of the alloyed CdS_*x*_Se_1−*x*_ NWs.

We further investigated the optical waveguide behavior of the achieved directional alloyed NWs. Figure 3c shows the real-color optical images of the NW with different S-molar fraction *x* under the excitation of focused laser. The big bright spot at excited position on the left is the in situ PL emission, which was guided through the NW and emitted at its end with a relatively weak emission (see the right spot of Fig. 3c). The emission spots are clearly seen at the right end of the NWs, indicating that as-grown directional NWs can form high-quality optical waveguide cavities as shown in Fig. 3c. It is noted that the emission color in the right end of the NW shows a red-shift due to the absorption–emission-absorption (A–E–A) mechanism during the propagation process [48]. These results demonstrate that the as-grown directional NWs are good optical waveguide cavities. The above optical characterizations demonstrate the superior optical capabilities and high crystallinity of the band gap-engineered CdSSe NWs to realize high-performance optoelectronic devices.

**Fig. 4:**
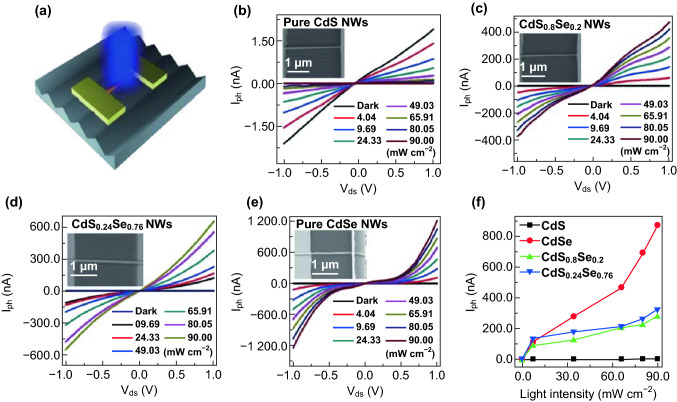
*I–V* curves of the in-plane-directional CdS_*x*_Se_1−*x*_ NWs. **a** Schematic diagram of the typical PD. **b**–**e** Representative *I–V* characteristics of the as-grown CdS_*x*_Se_1−*x*_ (*x* = 1, 0.8. 0.24, 0) directional NW PDs under dark and light illumination with different power intensities (laser wavelength: 405 nm) at room temperature; insets show the corresponding SEM images of the as-fabricated PDs. **f** Light intensity-dependent photocurrent values for the CdS_*x*_Se_1−*x*_ NW PDs

To this end, direct implementation of these directional NWs into photodetectors on the surface of sapphire is critical for their on-chip integration as schematically shown in Fig. 4a. The Cr/Au (10 nm/60 nm) electrodes are then deposited on the NWs (diameter 100 nm) by electron beam evaporation. The optoelectronic properties of the as-fabricated photodetectors were investigated by exploring their photocurrent generation, photoresponsivity, quantum efficiency, stability, and response speed at room temperature. In general, the significant increase in photocurrent occurs due to electron–hole pairs excited by the incident light intensity with energy larger than the band gap [49]. Here, the directional Cd_*x*_Se_1−*x*_ NWs can absorb maximum light in the visible region of the spectrum due to long wavelength range, indicating that the NWs could be easily excited by visible light. Figure 4b–e shows a representative set of current–voltage (*I*–*V*) curves for the directional Cd_*x*_Se_1−*x*_ (*x *= 1, 0.8, 0.24, 0) NWs under dark condition and illumination of 405 nm laser with different light intensities.

The linear shape of the *I*–*V* curves shows good ohmic contacts between the NWs and the electrodes for the as-constructed PDs consistent with the reported work on the free-standing nanostructures [50]. The *I-V* results show that the value of the photocurrents in dark condition is very small and enhances suddenly, for all the constructed photodetectors, under light illumination. Light intensity-dependent photocurrents were analyzed under the dark condition and 405 nm laser illumination with different intensities to compare CdS_*x*_Se_1−*x*_ PDs with different elemental composition. It can be seen that the PDs with different elemental compositions exhibit very different photocurrent responses. As the composition *x* value decreased from 1, 0.8, 0.24 to 0, the photocurrent value gradually increased from 1.89 nA for CdS to 1.2 µA for CdSe directional NWs, respectively, as shown in Fig. 4f. The observed photocurrent response to light illumination indicates an obvious increase in the number of photo-excited mobile charge carriers that result from the uprising of Fermi level of the carriers and demonstrates the successfully obtained PDs based on directional nanowire modulation bandgap from pure CdS to CdSe [49, 51].

**Fig. 5:**
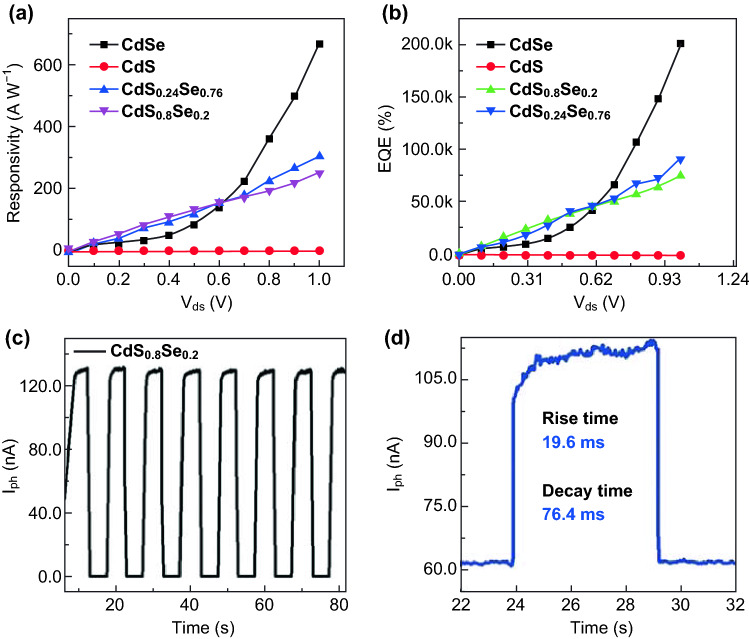
Photodetection properties of the directional CdS_*x*_Se_1−*x*_ NWs. **a** The responsivity of the CdS_*x*_Se_1−*x*_ nanowire PDs. **b** Plot of quantum efficiency of the CdS_*x*_Se_1−*x*_ PDs. **c** Time-resolved response of the device under light intensity 49.03 mW cm^−2^ at a voltage of 1 V. **d** On/off photocurrent response and corresponding enlarged portions of the rise in response and reset process of CdS_0.8_Se_0.2_ nanowires under excitation of 405 nm with a power intensity of 9.69 mW cm^−2^

Photoresponsivity (*R*) is one of the most important parameters for a PD and is expressed by *I*_ph_/*PS*, where *I*_ph_ is photocurrent, *P* and *S* are the incident power density and effective irradiated area on the device, respectively [52]. The calculated *R* values for the light intensity 90 mW cm^−2^ of the PDs are shown in Fig. 5a. From these calculated results, we found that for CdS_*x*_Se_1−*x*_ PDs (*x *= 1, 0.8, 0.24, 0), responsivity increases gradually on shifting from CdS to CdSe NW; as the bandgap energy decreases, a remarkable increase in the carrier concentration and consequent conduction in directional Cd_*x*_Se_1−*x*_ NWs occurs [36]. Compared to the binary CdSe, there is a compositional disorder in the ternary alloys with different composition in directional CdSSe NWs and the disorder is apparently enhanced because the coordination number varies from site to site to accommodate the varying valences of the atoms [53]. This leads to a high density of localized states tailing from the conduction and valence bands and thus a reduction of the current responsivity. Another sensitive parameter for NW PD is the external quantum efficiency (*EQE*) which is related to the number of electron–hole pairs excited by one absorbed photon and can be calculated using the equation, *EQE* = h*cR*/(eλ), where h is Planck’s constant, *c* is the velocity of light, e is the electronic charge, and λ is the incident light wavelength [2]. Figure 5b shows the calculated *EQE* of the as-grown directional Cd_*x*_Se_1−*x*_ NW based PDs. The obtained maximum *R* and *EQE* are 700 A W^−1^ and 2 × 10^5^%, respectively, which are higher than previously reported values based on random CdS, CdSe and CdSSe NWs as shown in Fig. S6. We also characterized the photocurrent dynamics of the directional Cd_*x*_Se_1−*x*_ PDs to study the stability and repeatability of the photocurrent by monitoring the current as a function of time under 405 nm light at 1 V bias as shown in Figs. 5c and S3. Rapid increase in current on turning on the light illumination is observed, while turning off the light results in a drastic decay down to its initial current. This result suggests that the PDs are highly stable when exposed to periodic light. The photocurrent rise time and reset time are key factors in determining the sensitivity of the PD to a fast-varying optical signal. The rise time was defined as the time needed to reach 90% of the photocurrent from dark current value after light illumination and the reset time was defined as the time needed to reach 10% of the photocurrent after switching off the light illumination. The rising and reset times were 19.6 and 76.4 ms, respectively, for CdS_0.8_Se_0.2_ as represented in Figs. 5d and S6. These results demonstrate that high-performance PDs are constructed based on the achieved band-gap engineered directional CdS_*x*_Se_1−*x*_ NWs. Furthermore, these results indicate that the combination of 1D morphologies with precisely adjustable properties in virtue of an alloying process provide a promising route to optimize these materials for practical applications.

## Conclusion

In this work, we demonstrated the synthesis of single crystalline ternary directional CdS_*x*_Se_1−*x*_ NWs, exhibiting graphoepitaxial growth along nanogrooves on *M*-plane sapphire. We studied the optical properties regarding tunable PL and waveguide emission of the directional NWs. The as-grown CdS_*x*_Se_1−*x*_ NWs reveal excellent band edge emission and optical waveguide properties. Moreover, PDs were constructed on individual aligned CdS_*x*_Se_1−*x*_ NWs. The devices illustrated relatively high photoresponsivity ~ 670 A W^−1^ and fast response speed ~ 76 ms. The band-gap-engineered directional CdS_*x*_Se_1−*x*_ NWs are suitable candidates for the field of optoelectronics as they operate in the visible region in a large wavelength scale. In addition, this approach could enable the production of semiconductors with highly controlled orientation and composition, appropriate for a wide range of applications, including LEDs, lasers, photovoltaic cells, and photonic and nonlinear optical devices.

## Electronic supplementary material

Below is the link to the electronic supplementary material.

Supplementary material 1 (PDF 499 kb)
