# Supplementary material for: Controllable Vapor Growth of Large-Area Aligned CdSxSe1−x Nanowires for Visible Range Integratable Photodetectors
Source: Nanomicro Lett. 2018 Jun 23;10(4):58. doi: 10.1007/s40820-018-0211-7 (PMC6199103; doi:10.1007/s40820-018-0211-7)
Supplement: Supplementary file 1 — Supplementary material 1 (PDF 499 kb) [file 40820_2018_211_MOESM1_ESM.pdf]

Supporting Information for

Controllable Vapor Growth of Large Area Aligned CdS_xSe_{1-x} Nanowires for Visible Range Integratable Photodetectors

Muhammad Shoaib[1], Xiaoxia Wang[1], Xuehong Zhang[1], Qinglin Zhang[1], Anlian Pan[1,*]

[1]Key Laboratory for Micro-Nano Physics and Technology of Hunan Province, State Key Laboratory of Chemo/Biosensing and Chemometrics, School of Physics and Electronics, Hunan University, Changsha, Hunan 410082, People's Republic of China

*Corresponding author. E-mail: anlian.pan@hnu.edu.cn (Anlian Pan)

Supplementary Figures and Table

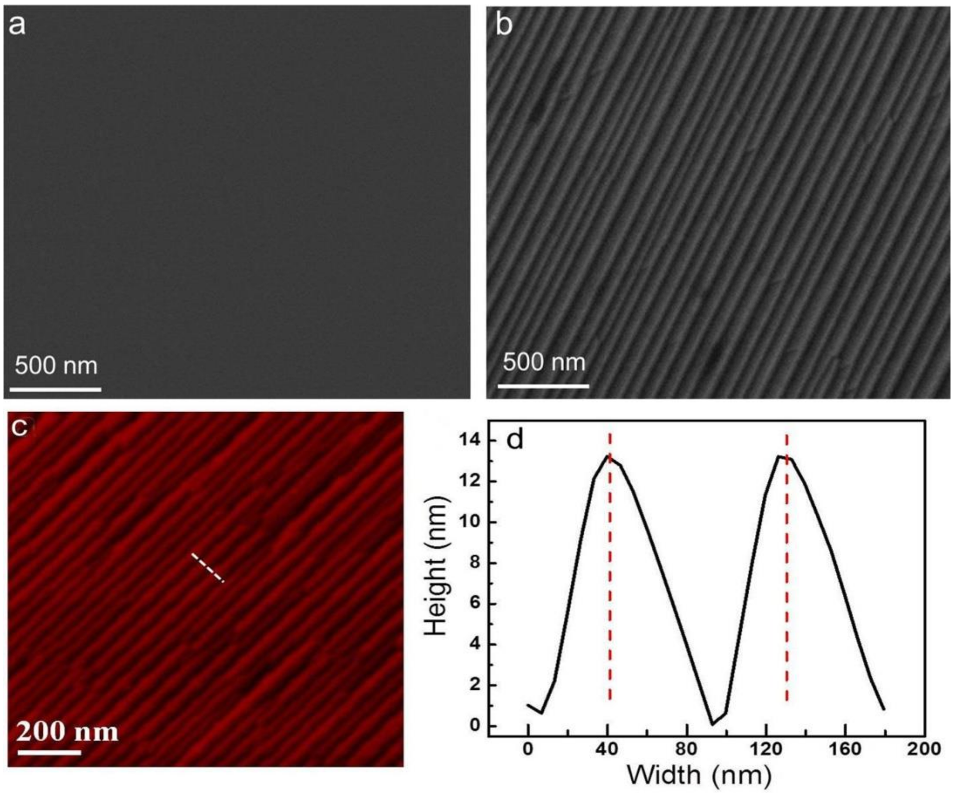

Fig. S1 Morphology characterization of the directional CdS_xSe_{1-x} NWs. **a** SEM image for the flat surface of the M-plane. **b** Realization of the faceted M-plane surface after high temperature annealing. **c** AFM image for the faceted M-plane substrate. **d** Height and width profile of the nanogrooves represents the dashed line marked position in **c**.

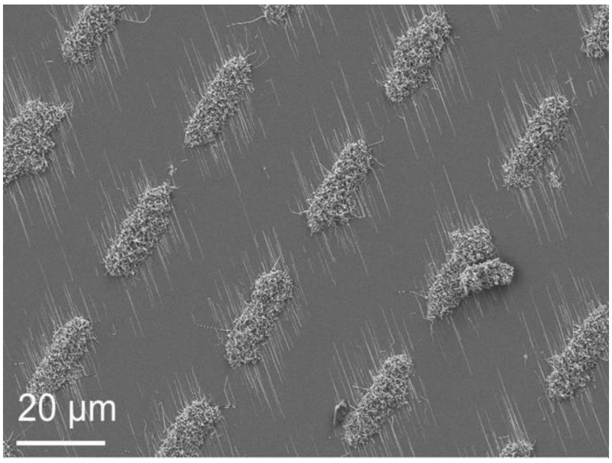

Fig. S2 Morphology characterization of the directional CdS_xSe_{1-x} NWs

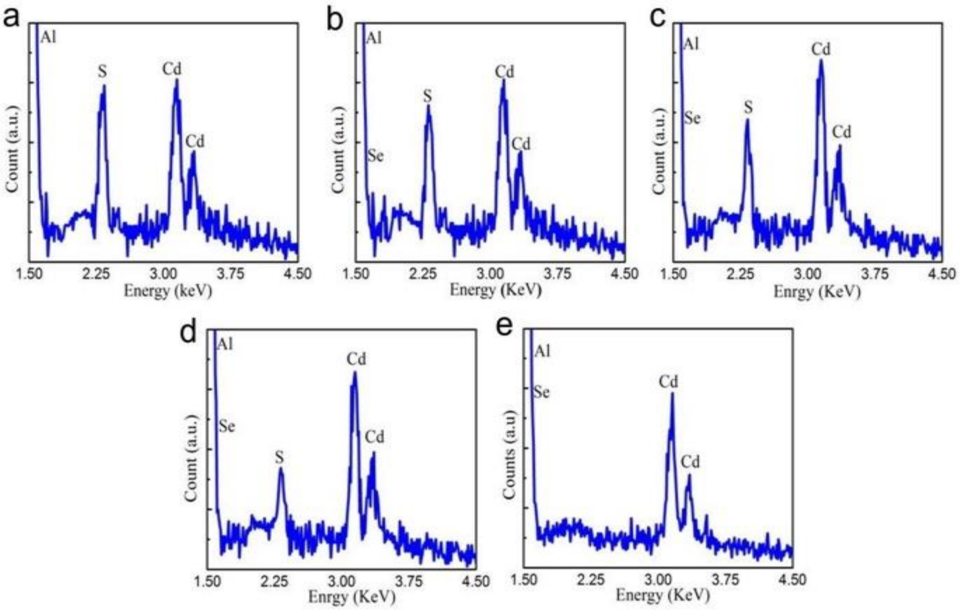

Fig. S3 EDS analysis of the CdS_xSe_{1-x} directional nanowires. **a-e** Elemental composition analysis from CdS to CdSe nanowires

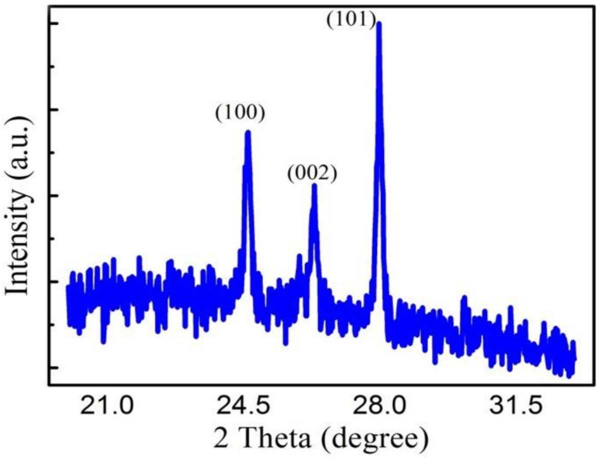

Fig. S4 XRD analysis of the CdS directional nanowires**. a** Wurtzite crystal structure graph for the directional pure CdS nanowires

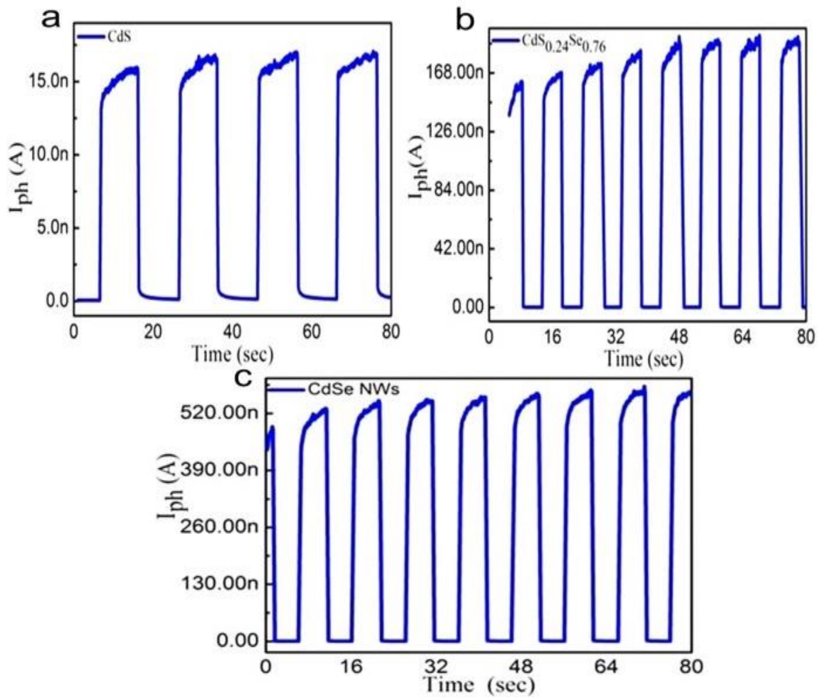

Fig. S5 Time resolved response of the photodectors. **a-c** On/off photocurrent response of the corresponding CdS, $CdS_{0.24}Se_{0.76}$ and pure CdSe directional nanowire respectively, under excitation of 405 nm with the power intensity of 9.69 Mw cm^{-2}

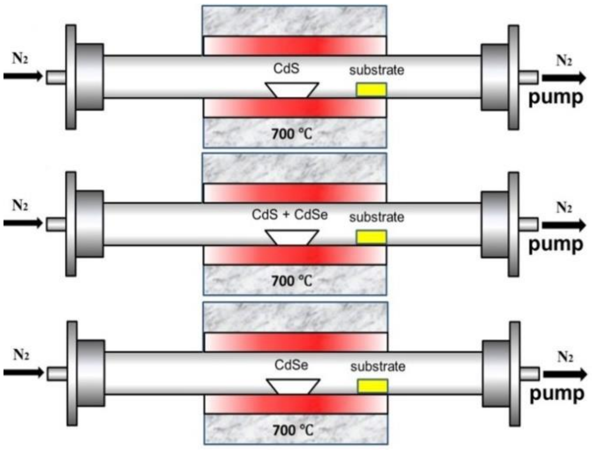

Fig. S6 Schematic setup for the growth of directional CdSSe NWs

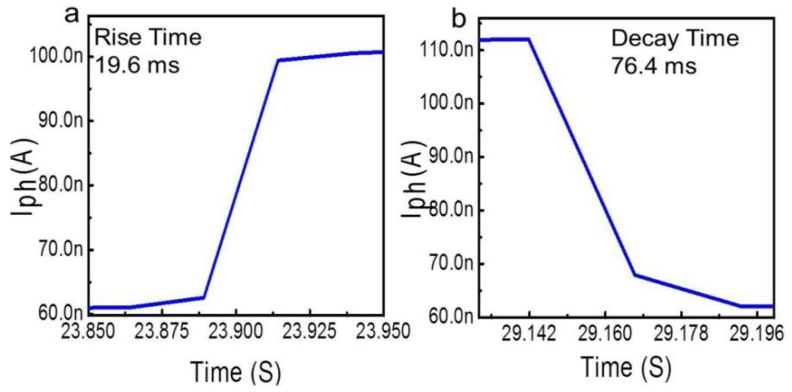

Fig. S7 Time resolved response of the photodectors. **a** Rise time graph, **b** Decay time graph

Table 1 The detailed properties comparison of the photodetectors in our work with the reported literatures

Photodetectors	*EQE*%	Responsivity (A W^{-1})	Rise Time (msec)	Decay Time (msec)	Ref.
CdS WA/CdSe SCS hetrostructures	3.25×10^3	13.1	1.4	2	1
Graded CdSSe NWs	-	-	-	240	2
CdS Nanobelts	-	-	1000	3000	3
Graded CdSSe NWs	-	1	-	-	4
CdS Nanobelt	2×10^2	5.2×10^2	0.137	0.379	5
CdSe NWs		0.3			6
CdSSe NWs	2×10^5	670	19.6	76.4	Our work

References

[1] G. Li, Y. Jiang, Y. Zhang, X. Lan, T. Zhai, G.-C. Yi, High-performance photodetectors and enhanced field-emission of CdS nanowire arrays on CdSe single-crystalline sheets. J. Mater. Chem. C **2**(39), 8252-8258 (2014). https://doi.org/10.1039/C4TC01503G

[2] T. Takahashi, P. Nichols, K.Takei, A.C. Ford, A. Jamshidi, M.C. Wu, C.Z. Ning, A. Javey, Contact printing of compositionally graded CdSxSe1−x nanowire parallel arrays for tunable photodetectors. Nanotechnology **23**(4), 045201(2012). https://doi.org/10.1088/0957- 4484/23/4/045201

[3] T. Gao, Q.H. Li, T.H. Wang, CdS nanobelts as photoconductors. Large-area photodetector with high-sensitivity and broadband spectral response based on composition-graded CdSSe nanowire-chip. Appl. Phys. Lett. **86**(17), 173105 (2005). https://doi.org/10.1063/1.1915514

[4] S. Guo, Z. Li, G. Song, B. Zou, X. Wang, R. Liu, J. Alloys Compounds **649**, 793-800 (2015). https://doi.org/10.1016/j.jallcom.2015.07.179

[5] Y. Ye, L. Dai, X. Wen, P. Wu, R. Pen, G.Qin, High-Performance Single CdS Nanobelt Metal-Semiconductor Field-Effect Transistor-Based Photodetectors. ACS Appl. Mater. Interfaces **2**(10), 2724-2727 (2010). https://doi.org/10.1021/am100661x

[6] E. Shalev, E. Oksenberg, K. Rechav, R. Popovitz-Biro, E. Joselevich, Guided CdSe nanowires parallelly integrated into fast visible-range photodetectors. ACS Nano **11**(1), 213-220 (2017). https://doi.org/10.1021/acsnano.6b04469
